# The Diagnostic Accuracy of the Nasopharyngeal Reflux Endoscopic Score (NRES) for Identifying Laryngopharyngeal Reflux Disease in Chronic Rhinosinusitis

**DOI:** 10.3390/jcm14124293

**Published:** 2025-06-17

**Authors:** Kalamkas Sagandykova, Nataliya Papulova, Gul’mira Muhamadieva, Talapbek Azhenov, Jerome R. Lechien

**Affiliations:** 1 “University Medical Center” Corporate Fund, School of Medicine, Nazarbayev University (NUSOM), Astana 010000, Kazakhstan; 2 Laryngopharyngeal Reflux Study Group, Young-Otolaryngologists of the International Federations of Oto-Rhino-Laryngological Societies (YO-IFOS), 13005 Paris, France; jerome.lechien@umons.ac.be; 3 Department of Otorhinolaryngology, NpJSC “Astana Medical University”, Astana 010000, Kazakhstan; papulova.n@amu.kz (N.P.); mukhamadieva.g@amu.kz (G.M.); azhenov.t@amu.kz (T.A.); 4 Department of Otolaryngology-Head and Neck Surgery, Foch Hospital, 78180 Paris, France; 5 School of Medicine, University Paris Saclay, 91190 Paris, France

**Keywords:** chronic rhinosinusitis with or without nasal polyps (CRSwNPs/CRSsNPs), laryngopharyngeal reflux disease, the Nasopharyngeal Reflux Endoscopic Score (NRES), the Reflux Symptom Index (RSI), the Reflux Symptom Score (RSS)

## Abstract

**Background**: Chronic rhinosinusitis with or without nasal polyps (CRSwNPs/CRSsNPs) is an inflammatory disease that is becoming increasingly associated with laryngopharyngeal reflux disease (LPRD). Although symptom-based questionnaires, such as the Reflux Symptom Index (RSI) and Reflux Symptom Score (RSS), are widely used, there is a lack of objective endoscopic tools for assessing the nasopharyngeal and nasal manifestations of reflux. The Nasopharyngeal Reflux Endoscopic Score (NRES) is a novel endoscopic scoring system that was developed to address this issue. Objective: The objective of this study was to evaluate the diagnostic accuracy of the NRES in identifying LPRD in patients with CRS, compared with a clinical reference standard. **Methods**: A prospective diagnostic accuracy cohort study was conducted at two tertiary care centers in Astana, Kazakhstan, from September 2023 to February 2025. A total of 216 adults were enrolled and divided into three groups: CRS with suspected LPRD (n = 116), CRS without LPRD (n = 69), and healthy controls (n = 31). CRS was diagnosed according to the EPOS 2020 criteria. LPRD was defined using a composite reference standard comprising clinical assessment, RSS > 13, RSI, and selective 24 h pH monitoring and gastrointestinal endoscopy. All participants underwent nasopharyngeal and laryngeal endoscopy, with NRES, L-K, RFS, RSI, and RSS assessments at baseline and at 6 and 12 months. Receiver operating characteristic (ROC) analysis was used to evaluate the diagnostic performance, and Wilcoxon tests were used to analyze the changes in scores. Correlation and regression analyses were used to explore associations between scales and predictive factors. **Results**: At baseline, NRES scores were significantly higher in the CRS with LPRD group (mean: 11.59) than in the CRS without LPRD group (mean: 3.10) and the healthy control group (mean: 2.16) (*p* < 0.001). ROC analysis demonstrated excellent diagnostic accuracy, with an area under the curve (AUC) of 0.998 (95% confidence interval (CI): 0.994–1.000), a sensitivity of 98% (95% CI: 94–100%) and a specificity of 96% (95% CI: 91–99%) at an optimal cut-off point of 8.5. NRES scores showed strong correlations with RSI, RSS, and RFS scores (r > 0.76, *p* < 0.001). A longitudinal assessment revealed significant reductions in all scores after treatment with proton pump inhibitors and lifestyle modifications, with sustained improvement at 12 months. Regression analysis found no significant effect of age, gender, or GERD severity (LA classification) on NRES scores. **Conclusions**: The NRES is a highly sensitive and specific endoscopic tool for identifying nasopharyngeal changes associated with LPRD in CRS patients. It demonstrates strong correlations with established symptom-based and laryngoscopic reflux assessments and responds to anti-reflux therapy over time. The NRES may, therefore, be a valuable objective adjunct in the comprehensive evaluation and longitudinal monitoring of LPRD-associated CRS.

## 1. Introduction

Chronic rhinosinusitis (CRS), with or without nasal polyps (CRSwNPs/CRSsNPs), is a multifactorial inflammatory condition affecting the nasal and paranasal sinuses. It is often resistant to conventional medical and surgical treatments [1,2,3]. There is an increasing body of evidence linking CRS with laryngopharyngeal reflux disease (LPRD), which is distinct from gastroesophageal reflux disease (GERD) because of its primary impact on the upper aerodigestive tract [3,4]. GERD has been linked to various extraesophageal manifestations, including laryngitis, chronic cough, asthma, otitis media with effusion, and, more recently, CRS. Studies estimate that up to 60% of CRS patients exhibit LPRD features, suggesting significant overlap [5,6,7].

The proposed mechanism by which LPRD contributes to CRS involves the direct exposure of the nasopharyngeal and sinus mucosa to refluxed gastric contents, including acid and pepsin. This exposure disrupts epithelial integrity, impairs mucociliary clearance, and sustains chronic inflammation [8,9]. Histopathological findings, including epithelial hyperplasia, goblet cell metaplasia, and inflammatory infiltration, further support reflux as a contributor to remodeling and dysfunction of the sinuses [9,10].

However, diagnosing LPRD-associated CRS remains clinically challenging because of the overlap of symptoms with those of allergic or infectious rhinitis and the limitations of current diagnostic tools. Objective methods, such as 24 h multichannel intraluminal impedance-pH (MII-pH) monitoring, assess esophageal reflux but provide limited insight into nasopharyngeal or sinus exposure [11,12]. Symptom-based tools, such as the Reflux Symptom Index (RSI) and Reflux Symptom Score (RSS), rely on subjective reporting and fail to capture observable mucosal pathology. Similarly, the Reflux Finding Score (RFS), a validated endoscopic tool for laryngeal signs of reflux, lacks specificity for nasopharyngeal and sinus involvement [13].

In order to address these diagnostic limitations, we developed the Nasopharyngeal Reflux Endoscopic Score (NRES), a standardized endoscopic tool designed to objectively evaluate changes to the nasopharyngeal mucosa associated with LPRD. The NRES evaluates parameters such as erythema, edema, mucus congestion, and mucosal granularity, which reflect reflux-induced inflammation that is often overlooked by esophageal-focused diagnostics. As an addition to the existing symptom-based and laryngeal assessment tools, the NRES aims to improve diagnostic accuracy, enable the early detection of reflux-related sinus and nasal disease, and inform personalized treatment strategies for CRS patients with suspected LPRD.

This study aims to evaluate the diagnostic accuracy of the NRES in identifying LPRD in patients with CRS using a composite clinical reference standard based on RSI, RSS, clinical assessment, and selective pH monitoring or endoscopy. We hypothesize that the NRES will demonstrate high sensitivity and specificity in distinguishing CRS associated with LPRD from CRS without LPRD and from healthy controls, thereby offering clinicians a reliable, objective tool.

## 2. Materials and Methods

The study protocol was approved by the Local Ethics Committee of Astana Medical University, Astana, Kazakhstan (Approval Number: LCB AMU #13). Written informed consent was obtained from all participants. The anonymized study dataset is publicly available at https://doi.org/10.6084/m9.figshare.28861565, accessed on 25 April 2025.

### 2.1. Study Design

This was a prospective, observational, comparative cohort study with longitudinal follow-up. It was conducted from September 2023 to February 2025 at two tertiary care centers in Astana, Kazakhstan: the University Medical Centre Corporate Fund (UMC CF) and Multidisciplinary Hospital No. 1.

### 2.2. Participants

Consecutive adults aged ≥18 years presenting with chronic nasal symptoms persisting for at least 12 months were screened for eligibility. The inclusion criteria included a diagnosis of chronic rhinosinusitis (CRS), with or without nasal polyps (CRSwNPs or CRSsNPs), based on the 2020 European Position Paper on Rhinosinusitis and Nasal Polyps (EPOS 2020) criteria [14]. This encompasses clinical symptoms, nasal endoscopy using the Lund–Kennedy (L-K) score, and computed tomography (CT) imaging findings. The exclusion criteria included recent nasal surgery (within six months), other diseases affecting the sinuses (e.g., tumors or granulomatous diseases), or an inability to provide informed consent. Healthy controls were age- and sex-matched volunteers without any symptoms of sinus disease or known reflux disease. The participants were stratified into three groups based on the reference standard for laryngopharyngeal reflux disease (LPRD):

CRS with LPRD (n = 116);
CRS without LPRD (n = 69);
Healthy controls (n = 31).
Reference standard for LPRD.

The reference standard for diagnosing LPRD was a composite assessment by an otolaryngologist and a gastroenterologist. This was based on a clinical evaluation and the following:

− The Reflux Symptom Score (RSS) with a cut-off of >13 [15].
− The Reflux Symptom Index (RSI) [16].
− Physician judgment incorporating symptom patterns and response to proton pump inhibitor (PPI) therapy. In cases where diagnosis was uncertain, 24 h dual-probe pH monitoring and gastrointestinal endoscopy were performed to confirm gastroesophageal reflux disease (GERD) and characterize the reflux phenotype. The assessors were blinded to the Nasopharyngeal Reflux Endoscopic Score (NRES) results.

Index test: Nasopharyngeal Reflux Endoscopic Score (NRES).

The NRES is a novel endoscopic scoring system designed to detect nasopharyngeal mucosal changes suggestive of extraesophageal reflux (see Table 1). It assesses the following signs: asymmetry in the nasal cavity; hypertrophy of the inferior turbinate; absence of mucus; hypertrophy of the nasopharyngeal wall and Eustachian tube opening; increased vascular pattern; presence of mucus; erythema; atrophic changes; and granulations or fibrotic changes. Each sign is scored as 0 (absent), 1 (moderately expressed), or 2 (severely expressed), and the total score is then summed. Standardized fiberoptic nasopharyngoscopy with digital photo documentation was performed at each visit. Two independent otolaryngologists, blinded to the patients’ symptom scores and clinical group, evaluated the images and resolved any discrepancies by consensus.

### 2.3. Data Collection

At baseline, all the participants underwent a comprehensive examination, including fiberoptic rhinoscopy with photo documentation, transnasal fiberoptic laryngoscopy, and assessments using the NRES, L-K score, and Reflux Finding Score (RFS). Symptom scores (RSS and RSI) were collected. Patients diagnosed with LPRD received 20 mg of omeprazole twice daily for one to two months after the visit, alongside lifestyle modifications to mitigate GERD symptoms. Follow-up assessments at six and twelve months involved repeating the baseline examinations. A second clinic visit within one week of each assessment ensured consistency.

### 2.4. Blinding

The assessors who determined the LPRD reference standard were blinded to the NRES results, and the otolaryngologists who scored the NRES were blinded to the clinical data and reference standard outcomes.

### 2.5. Statistical Methods

The diagnostic accuracy of the NRES was evaluated using a receiver operating characteristic (ROC) analysis of the baseline data. The area under the curve (AUC), as well as the sensitivity, specificity, positive predictive value (PPV), and negative predictive value (NPV), were calculated at an optimal cut-off point, which was determined using the Youden index. Between-group differences at baseline were assessed using the Kruskal–Wallis test, followed by Dunn’s test with Bonferroni adjustment for pairwise comparisons. Longitudinal changes in the LPRD group were analyzed using paired Wilcoxon tests with Holm’s correction at baseline and at the 6- and 12-month time points. Spearman’s rank correlation was used to assess the associations between the NRES, RSS, RSI, RFS, and L-K scores. Multiple linear regression evaluated the dependence of the NRES on age, gender, and GERD severity (Los Angeles classification). Participants with missing baseline data for the NRES or the reference standard were excluded from the analysis of diagnostic accuracy; the longitudinal analysis included only those with complete follow-up data. Analyses were performed using IBM SPSS Statistics 25, with *p* < 0.05 being considered significant.

## 3. Results

### 3.1. Participant Flow and Baseline Characteristics

Between September 2023 and February 2025, a total of 250 participants were screened for eligibility. Of these, 216 met the inclusion criteria and were enrolled: 116 had chronic rhinosinusitis (CRS) and laryngopharyngeal reflux disease (LPRD) (Group 1); 69 had CRS but not LPRD (Group 2); and 31 were healthy controls (Group 3). All the participants completed the baseline assessment, with no indeterminate results or loss to follow-up reported for the index test (Nasopharyngeal Reflux Endoscopic Score, NRES) or reference standard. Baseline demographic and clinical characteristics are summarized in Table 2.

CRS with nasal polyps: The number and percentage of participants with CRS and nasal polyps. For group 3 (healthy control participants), this indicator is not applicable (N/A), as they do not have CRS.

The Kruskal–Wallis H test was used to evaluate differences in reflux- and sinus-related indicators across the three groups, as this is a non-parametric method suitable for non-normally distributed data. Statistically significant differences were observed for the Reflux Symptom Score (RSS), Reflux Symptom Index (RSI), Reflux Finding Score (RFS), Nasopharyngeal Reflux Endoscopic Score (NRES), and Lund–Kennedy (L-K) score (all *p* < 0.0001).

**Figure 1:**
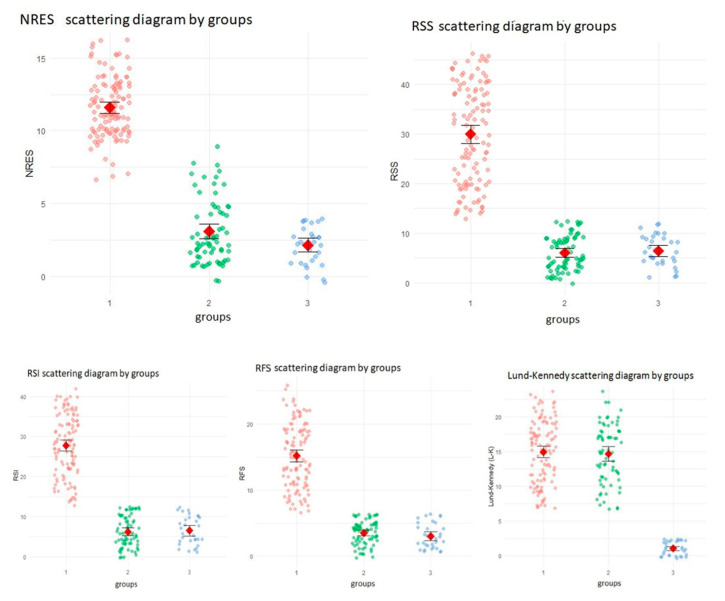
Boxplots of baseline scores across the study groups. NRES Scattering Diagram by groups. Figure 1 uses a scatter diagram to illustrate the distribution of baseline Nasopharyngeal Reflux Endoscopic Score (NRES) values across the three distinct study groups. The groups are defined as follows: Group 1: patients with chronic rhinosinusitis (CRS) and laryngopharyngeal reflux disease (LPRD); Group 2: patients with CRS without LPRD; and Group 3: healthy controls. This figure visually compares NRES scores to assess the diagnostic accuracy of the NRES in identifying LPRD among CRS patients. Each data point represents an individual NRES score, and summary statistics are highlighted for each group. Axes: The X-axis is labeled ‘groups’, with categories 1, 2, and 3 corresponding to the study groups described above. The Y-axis is labeled ‘NRES’, with a range from 0 to 15 representing the NRES score values. Visual elements: Colored dots: Individual NRES scores are plotted as colored dots. Pink dots represent scores for Group 1 (CRS with LPRD) and show a dense cluster ranging from approximately 5 to 15. Green dots represent scores for Group 2 (CRS without LPRD) and are distributed between 0 and 10. Blue dots represent scores for Group 3 (healthy controls), concentrated near 0 with values typically between 0 and 3. The distinct colors differentiate the groups, highlighting variations in NRES scores across the populations. Red diamonds indicate the mean NRES score for each group: Group 1 is positioned around 10–12, reflecting a higher average NRES score; Group 2 is positioned around 5–7, indicating a moderate average score; and Group 3 is positioned around 2–3, showing the lowest average score. Group-specific observations: Group 1 (CRS with LPRD) displays a broad range of NRES scores (5–15) and a higher mean (red diamond at 10–12), suggesting signs of elevated nasopharyngeal reflux consistent with LPRD. Group 2 (CRS without LPRD) shows a more moderate range (0–10), with an average score of around 5–7. This indicates that the NRES scores are lower than in Group 1. Group 3 (healthy controls) exhibits minimal NRES scores (0–3) with an average of around 2–3, reflecting an absence of significant reflux symptoms in healthy individuals.

Post hoc pairwise comparisons using Dunn’s test with Bonferroni adjustment revealed that Group 1 (CRS with LPRD) had significantly higher RSS, RSI, RFS, and NRES scores than Groups 2 (CRS without LPRD) and 3 (healthy controls) (all *p* < 0.0001). No significant differences were found between Groups 2 and 3 for these parameters (*p* > 0.05), suggesting that elevated reflux-related and nasopharyngeal findings were specific to the patients with CRS and LPRD. For the L-K score, no significant difference was observed between Groups 1 and 2 (*p* = 0.888), indicating comparable sinus inflammation in the CRS patients regardless of LPRD status. However, Groups 1 and 2 both had significantly higher L-K scores than Group 3 (*p* < 0.0001). Detailed distributions are shown in Figure 1.

The diagnostic accuracy of the NRES in identifying LPRD-associated CRS was assessed using a receiver operating characteristic (ROC) curve. This analysis included all 216 participants. Group 1 (CRS with LPRD, n = 116) was classified as ‘disease present’ (coded as 1), while Groups 2 (CRS without LPRD, n = 69) and 3 (healthy controls, n = 31) were classified as ‘disease absent’ (coded as 0). The area under the curve (AUC) was 0.998 (95% CI: 0.994–1.000), indicating exceptional discriminatory ability. At the optimal cut-off point of NRES ≥8.5 (as determined by the Youden index), the sensitivity was 98% (95% CI: 94–100%) and the specificity was 96% (95% CI: 91–99%). The positive predictive value (PPV) was 97% (95% CI: 93–99%), and the negative predictive value (NPV) was 97% (95% CI: 92–99%). These results suggest that the NRES is a reliable tool for identifying LPRD-associated CRS. The results are summarized in Table 3.

### 3.2. Longitudinal Changes in Scores

**Figure 2:**
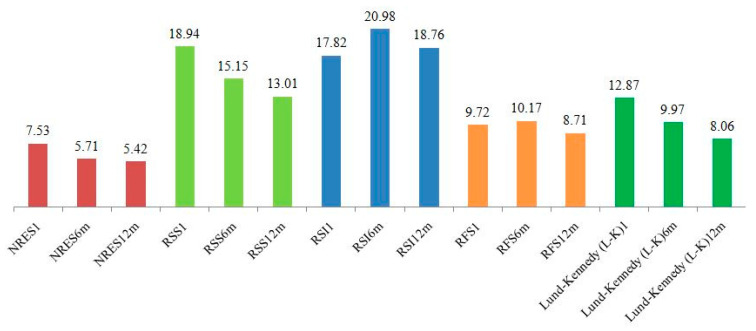
Longitudinal changes in scores at baseline, 6 months, and 12 months for the study groups. This figure presents a bar chart showing the mean values of various indicators for the three study groups at different time points after treatment application: baseline, six months, and 12 months. The indicators include the Nasopharyngeal Reflux Endoscopic Scale (NRES), the Reflux Symptom Index (RSI), the Reflux Symptom Score (RSS), the Reflux Findings Score (RFS), the Lund–Kennedy Scale (LKS), and the Los Angeles (LA) Classification for gastroesophageal reflux disease (GERD) severity. The groups are distinguished by column color, and the heights of the columns reflect the mean values. Axes: The X-axis shows the indicator categories and time points, including NRES1, NRES6m, and NRES12m, representing the NRES at baseline and at 6 and 12 months; RSS1, RSS6m, and RSS12m, representing the RSS at baseline and after 6 and 12 months; RSI1, RSI6m, and RSI12m, representing the RSI at baseline, after 6 months, and after 12 months; and LA1, LA6m, and LA12m, representing the LA classification at baseline and at 6 and 12 months. The Y-axis shows the mean values of the indicators, scaled from 0 to 25, with each value reflecting the mean group score. Color coding: Red: Group 1 (CRS and LFRH patients) at baseline (e.g., NRES1 = 7.5, RSS1 = 5.7). Green: Group 1 (CRS and LFRZ patients) at 6 and 12 months (e.g., NRES6m = 18.9, RSS6m = 21.0; NRES12m = 15.2, RSS12m = 17.8). Blue: Group 2 (CRS patients without LFRH) after 6 months (e.g., RSI6m = 18.8) and after 12 months (e.g., RSI12m = 13.0). Orange: Group 3 (healthy control subjects) after 6 months (e.g., RFS6m = 9.7) and after 12 months (e.g., RFS12m = 10.2). Green (additional shading): Group 3 after 12 months for certain indicators (e.g., L-K12m = 12.9 and LA12m = 10.0).

Changes in the NRES, RSS, RSI, RFS, and L-K scores were assessed in Group 1 (n = 116) at six and twelve months following treatment. Paired Wilcoxon tests with Holm’s correction revealed significant reductions in all scores from baseline to six months and from baseline to 12 months (all *p* < 0.001). Further improvements between six and twelve months were significant for the NRES (*p* = 0.017), RSS (*p* < 0.001), and L-K (*p* < 0.001), indicating sustained treatment effects. Figure 2 illustrates the dynamics.

### 3.3. Correlation Across Scales

Spearman’s correlation analysis was conducted to explore the relationships between the NRES, RSS, RSI, RFS, and L-K scores at baseline. Strong positive correlations were observed between the NRES and RSS (r = 0.768, *p* < 0.001), RSI (r = 0.766, *p* < 0.001), and RFS (r = 0.769, *p* < 0.001), which supports the convergent validity of the NRES. A weaker correlation was found with L-K (r = 0.221, *p* < 0.01), indicating that the NRES captures changes specific to reflux rather than general sinus inflammation. The results are presented in Table 4.

### 3.4. Regression Analysis

Multiple linear regression was performed to examine the relationship between the baseline NRES scores and clinical variables (age, race, Los Angeles (LA) classification of GERD severity) in Group 1 (n = 116). The model yielded an R^2^ of 0.061 (adjusted R^2^ = 0.018), indicating low explanatory power, and was not statistically significant (F = 1.419, *p* = 0.223). No predictors—age (*p* = 0.156), race (*p* = 0.272), or LA classification (*p* > 0.05)—were significantly associated with the NRES scores, though a trend toward lower scores in LA category C (*p* = 0.060) was noted. The results are detailed in Table 5.

## 4. Discussion

Chronic rhinosinusitis with or without nasal polyps and laryngopharyngeal reflux disease (LPRD) are interrelated conditions that may reinforce each other via shared pathological mechanisms. CRS is characterized by long-term inflammation of the nasal and paranasal mucosa, while LPRD occurs when gastric contents enter the upper respiratory tract and cause irritation and inflammation. Understanding these mechanisms is crucial for developing effective diagnostic and therapeutic strategies, such as using the Nasopharyngeal Reflux Endoscopic Score (NRES) [14]. Its pathophysiology is multifactorial, encompassing infectious, allergic, environmental, and, increasingly, reflux-related mechanisms [17]. Over the past two decades, extraesophageal reflux (EER)—the retrograde flow of gastric contents beyond the esophagus into the larynx, pharynx, nasopharynx, and, potentially, the sinus cavities—has gained attention as a contributor to upper airway disease, including CRS [18,19,20,21,22,23]. Unlike classical gastroesophageal reflux disease (GERD), EER can involve acidic or non-acidic refluxate, which can promote pathology by causing direct epithelial injury, mucociliary dysfunction, disruption to the epithelial barrier, and sustained mucosal inflammation [24,25,26,27,28,29].

CRS and LPRD reinforce each other through the following pathogenetic mechanisms:

Increased inflammation by reflux: Research suggests that when stomach acid and pepsin enter the nasopharynx following reflux, they activate inflammatory pathways. This increases the production of cytokines, such as IL-6 and TNF-α, which attract immune cells and exacerbate CRS, particularly in refractory cases [1,30,31]. Studies such as that by Liu et al. (2021) demonstrate how LPR can lead to inflammatory responses in the upper aerodigestive tract, including the sinuses, thus supporting this mechanism [30]. Lechien et al. (2023) further confirm that reflux contributes to inflammation in CRS, especially in recalcitrant cases [1].

The vicious circle of mucociliary dysfunction: It is thought that mucus stagnation in the sinuses of people with CRS can promote reflux, creating a cycle. In turn, reflux damages the ciliated epithelium, impairing the body’s ability to clear mucus and maintaining chronic inflammation and symptoms in both conditions. Prithviraj et al. (2024) discuss how LPR contributes to CRS through such mechanisms, emphasizing the cycle’s impact [32].

Epithelial vulnerability: There is evidence that reflux damages the epithelium, reducing its barrier properties and making the mucosa more susceptible to infections and allergens, thus exacerbating CRS [1,30]. Liu et al. (2021) directly address how LPR leads to mucosal barrier dysfunction, thereby increasing vulnerability to infections and allergens [30]. This aligns with the aforementioned mechanism. This damage creates a feedback loop that worsens CRS symptoms and inflammation.

Immune modulation: Studies indicate that reflux can enhance Th2-mediated inflammation, which is characteristic of CRSwNPs (CRS with nasal polyps), and this could contribute to polyp formation and chronicity. Zhang et al. (2019) [33] discuss the role of Th2 cytokines in chronic rhinosinusitis with nasal polyps (CRSwNPs), suggesting that reflux could influence this immune response; however, more research is needed to confirm any direct links. Reflux can modulate immune responses, particularly by enhancing Th2-mediated inflammation, which is characteristic of CRSwNPs, as noted by Zhang et al. (2019) [33]. This can contribute to polyp formation and the chronic nature of the disease.

Aldajani et al. (2024) provide evidence of an association between reflux diseases and CRS, supporting the potential for immune modulation [2]. However, direct links require further research.

The literature provides strong evidence of the pathogenetic mechanisms linking chronic rhinosinusitis (CRS) and laryngopharyngeal reflux disease (LPRD), including increased inflammation, mucociliary dysfunction, epithelial vulnerability, and immune modulation. These mechanisms create a reinforcing cycle that exacerbates both conditions, emphasizing the importance of tools such as NRES for diagnosis and management, as it detects the specific changes to the nasopharyngeal mucosa associated with reflux. NRES allows clinicians to objectively assess inflammation caused by LPRD and differentiate it from other causes of CRS. This information can inform treatment decisions, including the use of anti-reflux therapies, such as proton pump inhibitors, as well as lifestyle modifications, both of which could potentially improve patient outcomes.

However, diagnosing CRS associated with laryngopharyngeal reflux disease (LPRD) remains challenging because of symptom overlap with allergic rhinitis, non-allergic rhinitis, and primary CRS, which often leads to misdiagnosis or underdiagnosis [22,30]. Although symptom-based tools, such as the Reflux Symptom Index (RSI) and Reflux Symptom Score (RSS), are validated for quantifying reflux-related symptoms, their subjective nature limits their ability to assess mucosal changes directly [15,16]. Objective methods, such as 24 h esophageal pH monitoring and multichannel intraluminal impedance–pH (MII-pH) testing, are the gold standard for esophageal reflux [18,27], but they do not evaluate nasopharyngeal or sinus exposure. Similarly, the Reflux Finding Score (RFS), an endoscopic tool for laryngeal signs of reflux, lacks specificity for nasopharyngeal pathology [16].

In order to address this diagnostic gap, we developed the Nasopharyngeal Reflux Endoscopic Score (NRES), a standardized endoscopic tool for assessing nasopharyngeal mucosal changes linked to reflux. The NRES evaluates parameters such as erythema, edema, mucus congestion, and granularity, which are markers of chronic irritation and the epithelial response to reflux exposure. The primary aim of this study was to evaluate the diagnostic accuracy and clinical utility of the NRES in identifying laryngopharyngeal reflux disease (LPRD)-associated chronic rhinosinusitis (CRS). We hypothesized that the NRES would demonstrate high sensitivity and specificity in distinguishing CRS cases with nasopharyngeal reflux from other phenotypes. Our findings confirmed this, showing that the NRES is a reliable and reproducible tool for detecting LPRD-related nasopharyngeal changes.

### 4.1. Clinical Applicability of the NRES

The NRES is intended to complement existing diagnostic tools for CRS patients with suspected LPRD. Its ability to objectively assess nasopharyngeal inflammation enhances diagnostic precision, particularly in cases where symptoms are non-specific or do not respond to standard CRS treatments. In clinical practice, the NRES can be incorporated into the diagnostic process for patients presenting with symptoms such as persistent nasal obstruction, postnasal drip, or throat discomfort that suggest LPRD. By providing a standardized scoring system, the NRES enables consistent documentation of endoscopic findings and supports targeted therapeutic strategies, such as anti-reflux therapy. This could improve patient outcomes by identifying those who are likely to benefit from reflux management and reducing the need for prolonged empirical treatments.

Implementing the NRES requires fiber-optic nasopharyngoscopy and otolaryngologists who are trained in the scoring criteria. While this may present challenges in settings with limited resources, the potential for improved diagnostic accuracy and personalized treatment justifies efforts to increase its use. As this study was conducted in Kazakhstan, it remains to be confirmed whether the NRES can be generalized to other populations and healthcare systems. Validation in diverse demographic and clinical contexts would strengthen its applicability.

### 4.2. Limitations and Future Directions

A significant limitation of this study is the absence of hypopharyngeal–esophageal MII-pH monitoring, which is the gold standard for confirming LPRD. While MII-pH is effective at detecting esophageal reflux, it does not assess nasopharyngeal exposure. This highlights the need for complementary tools like the NRES. Future research should incorporate MII-pH to validate the NRES against this standard and explore correlations between esophageal and nasopharyngeal reflux events. Furthermore, larger multicenter studies are required to evaluate the performance of the NRES across different populations and refine its scoring parameters to achieve optimal sensitivity and specificity. Although research supports the association between CRS and LPRD, the exact mechanisms of their interaction remain poorly understood. For instance, the impact of non-acid reflux on the nasopharyngeal microbiome requires further investigation. Furthermore, studies are needed to determine whether early detection of reflux using tools such as the NRES can prevent the progression of CRS or reduce the need for surgical intervention.

## 5. Conclusions

The NRES is a significant advance in the diagnosis of LPRD-associated CRS, providing an objective and reproducible method that fills the gap left by existing tools. Its clinical applicability lies in enhancing diagnostic accuracy, guiding treatment decisions, and improving outcomes for patients with reflux-related upper airway disease. As further validation refines its utility, the NRES has the potential to become a cornerstone in the management of this challenging condition.

## Figures and Tables

**Table 1:** Nasopharyngeal Reflux Endoscopic Score (NRES).

Signs of Nasopharyngeal Reflux Exposure	0 (Absent)	1 (Moderately Expressed)	2 (Severely Expressed)
**Nose**			
Asymmetry between the anterior and posterior regions of the nasal cavity	☐	☐	☐
Predominantly unilateral hypertrophy of the posterior end of the inferior turbinate	☐	☐	☐
Absence of mucus in the middle nasal passage	☐	☐	☐
**Nasopharynx**			
Hypertrophy of the posterior wall of the nasopharynx	☐	☐	☐
Hypertrophy of the Eustachian tube opening	☐	☐	☐
Increased vascular pattern	☐	☐	☐
Presence of mucus	☐	☐	☐
Erythema or inflammation of the nasopharyngeal mucosa	☐	☐	☐
Atrophic changes in the mucosa	☐	☐	☐
Presence of granulations or fibrotic changes	☐	☐	☐

Table 1 shows the Nasopharyngeal Reflux Endoscopic Score (NRES), which is a standardized system for evaluating signs of nasopharyngeal reflux detected during an endoscopic examination. These signs are categorized by anatomical location as either ‘nose’ or ‘nasopharynx’. Each sign is graded according to its severity and given a score from 0 to 2, where 0 (absent) indicates that the sign is not observed; 1 (moderately pronounced) indicates that the trait is present but not pronounced; and 2 (strongly pronounced) indicates that the trait is clearly or significantly pronounced. The checkmarks (☐) under each score (0, 1, or 2) show the possible scores for each feature. The appropriate score is selected based on endoscopy data. The total NRES score is calculated by adding together the scores for all the individual features in order to determine the overall severity of nasopharyngeal reflux.

**Table 2:** Baseline characteristics of the study participants.

Characteristic	Group 1: CRS with LPRD (n = 116)	Group 2: CRS Without LPRD (n = 69)	Group 3: Healthy Controls (n = 31)
Age, mean (SD), years	45.2 (12.3)	44.8 (11.9)	43.5 (10.7)
Female, n (%)	68 (58.6%)	39 (56.5%)	18 (58.1%)
CRS with nasal polyps, n (%)	52 (44.8%)	30 (43.5%)	N/A

Table 2 shows the baseline demographic and clinical characteristics of the study participants, divided into three groups. Group 1: CRS with LFRH (n = 116), including the participants diagnosed with both chronic rhinosinusitis (CRS) and laryngopharyngeal reflux disease (LPRD). Group 2: CRS without LFRZ (n = 69), including the participants diagnosed with CRS but not LPRD. Group 3: Healthy control participants (n = 31), including healthy individuals who did not have a diagnosis of CRS or LFRZ and who served as the control group. The table below shows the characteristics of each group. Age (mean (SD)) in years: The mean age of the participants in each group, where the standard deviation (SD) shows the variation in ages within the group. Women, n (%): The number and percentage of women in each group.

**Table 3:** Diagnostic accuracy of the NRES at the optimal cut-off (NRES ≥ 8.5).

Metric	Estimate (95% CI)
AUC	0.998 (0.994–1.000)
Sensitivity	98% (94–100%)
Specificity, True Negative Rate, TNR	96% (91–99%)
Positive Predictive Value	97% (93–99%)
Negative Predictive Value	97% (92–99%)

Table 3 contains the key metrics for the diagnostic accuracy of the Nasopharyngeal Reflux Endoscopic Score (NRES), with an optimal threshold value of 8.5 or above. These metrics evaluate the NRES’s ability to detect laryngopharyngeal reflux (LPRD) in patients with chronic rhinosinusitis (CRS).

**Table 4:** Spearman correlation matrix.

	NRES	RSS	RSI	RFS	Lund–Kennedy (L-K)
NRES	1.000	0.768 *	0.766 *	0.769 *	0.221 *
RSS	0.768 *	1.000	0.787 *	0.729 *	0.242 *
RSI	0.766 *	0.787 *	1.000	0.758 *	0.278 *
RFS	0.769 *	0.729 *	0.758 *	1.000	0.284 *
Lund–Kennedy (L-K)	0.221 *	0.242 *	0.278 *	0.284 *	1.000

* Correlation is significant at the 0.01 level. Table 4 presents the Spearman correlation matrix demonstrating the relationships between the various indices measured at the start of this study. These include the Nasopharyngeal Reflux Endoscopic Scale (NRES), the Reflux Symptom Score (RSS), the Reflux Symptom Index (RSI), and the Reflux Findings Score (RFS), as well as the Lund–Kennedy (L-K) scale. The matrix shows the strength and direction of the associations between these scales, which is important for understanding their interrelationships and the diagnostic value of the NRES.

**Table 5:** Regression results.

Term	Estimate	Std. Error	Statistic	*p* Value
(Intercept)	12.94621936	0.766071047	16.89950222	0.000
age	−0.02046886	0.014314153	−1.429973523	0.156
race	−0.452673187	0.40994756	−1.104222178	0.272
LA Classification B	−0.153630492	0.432067518	−0.355570567	0.723
LA Classification C	−1.023346692	0.537950794	−1.902305385	0.060
LA Classification D	−0.860190641	1.46213281	−0.588312248	0.558

Table 5 shows the results of a multiple linear regression analysis that was carried out to evaluate the correlation between the initial scores on the Nasopharyngeal Reflux Endoscopic Scale (NRES) and various clinical factors in Group 1 (patients with chronic rhinosinusitis and laryngopharyngeal reflux, n = 116). The model included the following predictors: age, race, and the Los Angeles Classification (LA Classification) for the severity of gastroesophageal reflux disease (GERD). The model’s overall R^2^ was 0.061 (adjusted R^2^ = 0.018), indicating low explanatory power. The model, as a whole, was not statistically significant (F = 1.419, *p* = 0.223).

## Data Availability

The data presented in this study are openly available in https://doi.org/10.6084/m9.figshare.28861565 (accessed on 25 April 2025).

## Abbreviations

The following abbreviations are used in this manuscript:

CRSwNP/CRSsNP	chronic rhinosinusitis with or without nasal polyps
LPRD	laryngopharyngeal reflux disease
RSI	Reflux Symptom Index
RSS	Reflux Symptom Score
NRES	Nasopharyngeal Reflux Endoscopic Score
L-K	Lund–Kennedy
EPOS	European Position Paper on Rhinosinusitis and Nasal Polyps 2020
RFS	Reflux Finding Score
ROC	receiver operating characteristic
PPIs	proton pump inhibitors
GERD	gastroesophageal reflux disease
MII-pH	multichannel intraluminal impedance–pH
EER	extraesophageal reflux
